# Herd immunity: could it bring the COVID-19 pandemic to an end?

**DOI:** 10.2217/fmb-2020-0293

**Published:** 2021-04-13

**Authors:** Thomas Hugh Pennington

**Affiliations:** ^1^Aberdeen, UK

**Keywords:** antivaccinationists, asymptomatic, COVID-19, fatalities, herd, immunization, rinderpest, second wave, smallpox, transmission

The first comprehensive definition and detailed discussion of herd immunity appeared in 1929 in the first edition of the classic text, Topley and Wilson’s ‘The Principles of Bacteriology and Immunity’ [1]. The definition was broad, encompassing the innate resistance of a species to infection by a particular pathogen; herd habits and environmental and ecological factors promoting nonspecific immunity; as well as resistance acquired following infection or after immunization. They pointed out that freedom from a disease is not synonymous with specific immunity, saying that “*there is little doubt that the English herd, as such, is immune to plague and to typhus*” because plague rats are very rare and lousiness is very uncommon, but “*that is quite clear that the individuals who compose the English herd enjoy this immunity only so long as they remain within it.*” Nowadays, it is likely that it would have been considered wise to discuss this phenomenon with respect to the herds of other UK nations; the outbreak of yellow fever in Swansea in 1865 [2] would have been appropriate. The 29 cases with a fatality rate of 68% were transmitted by *Aedes aegypti* that had travelled from Cuba on the copper ore-carrying ship *Hecla*. This mosquito cannot breed in Wales, or anywhere else in the UK, because the winters are too cold, so it is not possible for the virus to become established.

As soon as it was coined, the use of the term ‘herd’ to designate human communities was criticized. Dudley [3] mounted a vigorous defence, also in 1929. He pointed out that it was first popularized by psychologists with the phrase ‘herd instinct’, and that biologically there is little fundamental difference between a herd of deer, a herd of swine, or a herd of *Homo sapiens*. Presciently, he also discussed the subherd of antivaccinationists. “*They form a very difficult administrative problem. They are perfectly honest in their convictions, but their power of dissociation and rationalization is so great that they often seem to the saner members of the herd to be absolutely unscrupulous and dishonest, whereas really they are only completely inaccessible to logic… making the most absurd accusations against those who dare to differ from them.*”

Considerations this year about COVID-19 control have in general used the term ‘herd immunity’ with a much narrower meaning than Topley’s and Wilson’s. They carry the implication of a level of specific immunity against the virus that will reduce the effective reproductive rate R_0_ to <1, so that the virus will not be able to maintain itself and continue to spread, a situation which should eventually result in its elimination. The calculation underpinning this view is [4]:

(Eq. 1) $${\text{p}}_{\text{c}}=1-(1/{\text{R}}_{0})$$

In which p_c_ is the proportion of the population that is immune, either as a consequence of an infection or by immunization with a vaccine or both [4]. It is reasonable to suppose that the term was used in this sense by Dr David Halpern, a government scientific advisor, when he said on 11 March 2020 on the BBC Today programe that “*You will want to protect those at risk groups so that they basically do not catch the disease and by the time that they come out of their cocooning, herd immunity has been achieved in the rest of the population.*” This strategy has been endorsed by the Great Barrington Declaration signed on 4 October 2020 by some infectious disease epidemiologists and public health scientists [5]: “*We know that all populations will eventually reach herd immunity, in other words, the point at which the rate of new infections is stable and that this can be assisted by (but is not dependent upon) a vaccine. Our goal should, therefore, be to minimize mortality and social harm until we reach herd immunity. The most compassionate approach that balances the risks and benefits of reaching herd immunity, is to allow those who are at minimal risk of death to live their lives normally to build up immunity to the virus through natural infection, while better protecting those who are at highest risk.*” Other scientists and healthcare professionals disagree profoundly with this strategy. Their views have been outlined in the John Snow Memorandum, first published on 14 October 2020 [6]: “*The arrival of a second wave and the realization of the challenges ahead has led to renewed interest in a so-called herd immunity approach, which suggests allowing a large uncontrolled outbreak in the low-risk population, while protecting the vulnerable. Proponents suggest this would lead to the development of infection-acquired population immunity in the low-risk population, which will eventually protect the vulnerable. This is a dangerous fallacy unsupported by scientific evidence…uncontrolled transmission in younger people risks significant morbidity and mortality across the whole population, furthermore, there is no evidence for lasting protective immunity to SARS-CoV-2 following natural infection.*”

A wide range of estimates of the herd immunity threshold for COVID-19 have been published. Formula (1) above assumes that everyone in the population is equally likely to become infected. However, there is abundant evidence that this is not the case. Social activity levels and age-related effects mean that the population is heterogeneous regarding the likelihood of becoming infected. Together with the large number of asymptomatic cases, these things make estimating the threshold a difficult task at this time [7,8].

It is often claimed that the eradication of smallpox testifies to the success of a herd immunity policy. It does not. The comment of Anderson and May [4] in this regard is appropriate: “*Too many of the putative facts known to public health planners rest on enthusiastic retelling of plausible tales, rather than on controlled experiments or careful analysis of data.*” On the basis of estimated values of its effective reproductive rate they calculated the degree of herd immunity required for smallpox eradication(p_c_) to be 70–80%. Before 1967, the WHO smallpox eradication programe was defined in terms of the number of vaccinations performed: “*It has been demonstrated that eradication of smallpox from an endemic area can be accomplished by vaccinating 80% of a population within a period of 4–5 years.*” However, it was found that outbreaks still occurred in districts where the 80% goal had been reached; in 1973 the goal had been achieved in India, but in that year it had 88,114 cases. Continued virus transmission in mass vaccinated communities was strongly associated with high population densities [9]. Accordingly, the WHO began to move to and implement a surveillance-containment strategy, finding that this was effective in controlling transmission, even when vaccination coverage was much less than 80%.

To date, rinderpest is the only infectious disease that has been eradicated by engendering herd immunity sufficient to lead to its extinction in the wild. Related to measles, it causes a disease in cattle and buffalo often with high morbidity and mortality. Historically, attempts to control it in Britain have an uncanny similarity to current events. Imported from Europe in 1865, it killed at least 420,000 cows [10]. Experts were hired. A Royal Commission (equivalent to a public inquiry today) was established. There was a debate about disinfectants. The contacts of infected animals were slaughtered. Railway transit stopped and fairs and markets were closed. Some members of the Commission dissented from these measures, saying that cattle movement stoppage, “*would involve an interference with the course of trade at variance with our national habits; and it would demand sacrifices from large numbers of people, who are removed from the presence of the disease.*” Legislation giving effect to control measures was hurried through the House of Commons in a manner that was described as savoring of despotism [11].

The UN Food and Agriculture Organization and the World Organization for Animal Health declared the global eradication of rinderpest in 2011. Absolutely central to this success was TRCV, the tissue culture rinderpest vaccine, which protected against all rinderpest variants, provided life-long immunity, was never associated with any adverse reactions, gave immunity after a single dose and in its ThermoVax form was thermostable, with a shelf life of 30 days outside the cold-chain, holding a maximum titre for 14 days at 45°C [12].

It is too early to tell whether any of the COVID-19 vaccines under development will possess all or even just some, of these properties. To block the transmission of SARS-CoV-2 by creating the necessary levels of herd immunity, much depends on vaccine efficacy and the duration of vaccine protection. For a vaccine with 100% efficacy that gives life-long protection, and taking into account prelockdown values of R_o_ of between 2.5 and 3.5, the level of herd immunity needed to block virus transmission is about 60–72% [13]. Less efficacious vaccines and ones that only give a short duration of protection will require a higher proportion of the population to be immunized to interrupt virus transmission and repeated vaccinations may be required, particularly if virus variants to which current vaccines offer poor protection become common.

However effective COVID-19 vaccines turn out to be, it is likely in most countries that the first batches will be mainly used to protect groups of individuals with the highest case fatality rates, in other words, those older than 70 years. It seems likely that herd immunity strategies will have to wait, giving time, perhaps, to counter the influence of the antivax sub herd and to address the issues that cause vaccine hesitancy.
